# Psychological Correlates of Problematic Gaming Behaviour in Medical Students: A Cross-Sectional Analysis of Structural and Contextual Factors

**DOI:** 10.3390/healthcare14152348

**Published:** 2026-08-01

**Authors:** Liliana Veronica Diaconescu, Alexandra Ioana Mihăilescu, Miruna Ioana Drăgușoiu, Ovidiu Popa-Velea

**Affiliations:** 1 Department of Medical Psychology, Faculty of Medicine, “Carol Davila” University of Medicine and Pharmacy, 020021 Bucharest, Romania; liliana.diaconescu@umfcd.ro (L.V.D.); miruna-ioana.dragusoiu0720@stud.umfcd.ro (M.I.D.); ovidiu.popa-velea@umfcd.ro (O.P.-V.); 2 Department of Psychiatry, “Prof. Dr. Alexandru Obregia” Clinical Hospital of Psychiatry, 041914 Bucharest, Romania

**Keywords:** internet gaming disorder, problematic gaming behaviour, medical students, conscientiousness, psychological distress, academic well-being

## Abstract

**Highlights:**

**What are the main findings?**

- A cut-off score of 24 on the Internet Gaming Disorder Scale—Short Form (IGDS9-SF) was associated with problematic gaming behaviour in 10.3% of students from a Romanian medical university.
- Higher daily gaming time, preference for competitive games and lower conscientiousness were the main independent predictors of higher IGDS9-SF scores.

**What are the implications of the main findings?**

- In addition to gaming duration, game type and self-regulation traits should be taken into account for screening problematic gaming in medical students.
- Prevention strategies could target time management, self-control and coping skills within stressful medical educational settings.

**Abstract:**

**Background**: Internet Gaming Disorder has drawn more attention as a public health problem in young adults; however, there are insufficient data regarding problematic gaming behaviour (PGB). This study explored the prevalence and predictors of PGB in students from a Romanian medical university. **Methods**: We implemented a cross-sectional survey of 243 Romanian and international undergraduate students from the University of Medicine and Pharmacy “Carol Davila” Bucharest. PGB was measured using the Internet Gaming Disorder Scale—Short Form. Personality traits, depressive symptoms, anxiety symptoms, perceived social support and prosocial behaviour were assessed by standardised instruments. Hierarchical multiple regression was used to identify predictors of the IGDS9-SF scores. **Results**: Based on a cut-off score of 24 on the IGDS9-SF, 10.3% of the participants were classified as having PGB (mean IGDS9-SF = 14.99, SD = 6.03). The final regression model accounted for 28.9% of the variance in IGDS9-SF scores (R^2^ = 0.289, *p* < 0.001), with gaming-related variables accounting for the largest incremental variance. Higher daily gaming time (*β* = 0.292, *p* = 0.001), competitive preferred game type (*β* = −0.232, *p* = 0.006), and lower conscientiousness (*β* = −0.201, *p* = 0.017) were significant independent predictors. IGDS9-SF scores were greater for competitive games. Perceived social support and prosocial behaviour did not show significant independent associations to PGB. Equally, depressive and anxiety symptoms improved model fit, but were not independent predictors. **Conclusions**: Among medical students, PGB demonstrates significant associations with gaming engagement and self-regulation, suggesting that screening and preventive approaches targeting these domains may represent relevant strategies for early identification and risk reduction.

## 1. Introduction

Videogaming, defined as “the experiential and interactive engagement facilitated by electronic games” [1], has become one of the most prominent forms of leisure and digital entertainment among younger populations worldwide [2]. Beyond its recreational dimension, videogaming represents a complex sociocultural and technological phenomenon that integrates cognitive stimulation, emotional involvement, and interpersonal interaction within digitally mediated environments. Contemporary video games encompass a broad spectrum of formats and genres, including single-player, cooperative, and competitive multiplayer modalities, each characterized by distinct patterns of participation, communication, and social engagement. These gaming environments often promote immersive experiences through reward systems, narrative structures, real-time interaction, and opportunities for social connectedness, thereby increasing their appeal among adolescents and young adults. As a result, the global videogame industry has experienced substantial growth in recent decades, further accelerated by the COVID-19 pandemic—during which social isolation and restricted mobility increased engagement with digital entertainment—with video gaming also serving as a means of facilitating social connectedness, managing stress, and reducing experiences of loneliness [3]. Current projections suggest further expansion of the industry, driven by rapid technological innovation and the integration of videogaming into broader educational, professional, and social domains. In particular, advances in virtual and augmented reality technologies, the increasing use of simulation-based training in medical and healthcare education, and the expanding global influence of electronic sports (Esports) are expected to further consolidate the role of videogaming in contemporary society [4].

In terms of public perception, videogaming is increasingly conceptualized not only as entertainment, but also as a multifaceted digital activity, with significant psychological, social, educational, and economic implications, a view supported by a growing body of evidence documenting its potential benefits. Reported positive outcomes include enhanced creativity, neuroplasticity, and specific memory functions, as well as improvements in problem-solving capacities and spatial cognition [5]. Additional research has identified beneficial effects on mood and emotional regulation [6], increased levels of physical activity [7], and greater self-efficacy and social competence. Furthermore, videogaming has been associated with the facilitation of social networking and the development of interpersonal relationships [8,9].

Nevertheless, despite these undeniable advantages, concerns persist regarding the potential adverse consequences of excessive video game engagement. In particular, children, adolescents, and young adults are considered especially vulnerable to the development of problematic gaming behaviour (PGB) and video game addiction. In particular, online gaming can lead to a pattern of problematic or compulsive behaviour. Recognizing the potential risks associated with excessive gaming, Diagnostic and Statistical Manual of Mental Disorders (5th Edition) included Internet Gaming Disorder (IGD) in Section 3 as a condition warranting further study [10,11]. Subsequently, IGD was formally recognized as a diagnosable condition in the International Classification of Diseases (11th Revision) [12]. Both diagnostic frameworks emphasize the importance of distinguishing normative gaming behaviour from maladaptive or pathological patterns of use. They also highlight the relevance of therapeutic interventions for excessive gaming behaviours, while underscoring the necessity of differentiating recreational engagement from problematic use.

Recent estimates suggest that more than two billion individuals worldwide engage in video gaming [13,14]. Among these, approximately 60 million individuals are thought to meet criteria associated with video game addiction [15,16], the majority of whom are adolescents and young adults. Current evidence indicates that the prevalence of IGD among young people ranges between 6.7% and 9.9% [17,18]. However, substantial regional variability has been reported, with estimates ranging from 1.2% to 15%, and being generally lower in Western countries and higher in Asian populations [19,20,21,22]. These differences may be partially explained by variations in diagnostic criteria and assessment methods, as well as by the increasing global accessibility of the internet and the broad adoption of smartphones and tablet devices [18].

In the academic environment, a previous meta-analysis reported a pooled prevalence of internet addiction of 30.1%, representing nearly a fivefold increase relative to the general population, while the pooled prevalence of IGD specifically was estimated at 6.2%, approximately twice that observed in the broader population [23]. This latter finding is particularly noteworthy, given that IGD diagnosis requires the endorsement of at least five DSM-5 criteria, suggesting that subclinical gaming-related difficulties may be considerably more prevalent in this particular category.

Although several theoretical frameworks emphasize different aspects of Internet Gaming Disorder (IGD), the present study focused on selected factors that may contribute to problematic videogaming behavior. Specifically, we drew on the Player’s Multidimensional Wellbeing (PMDWell) framework proposed by Olejarnik and Romano (2025), which encompasses social functioning, mental health, physical health, and life circumstances, with particular attention to the social functioning and mental health domains [24]. Within these domains, we distinguished between relatively stable, enduring factors (conceptualized as structural factors) and more dynamic, flexible factors that are likely to vary across contexts (conceptualized as contextual factors).

### 1.1. Factors Associated with Videogaming

#### 1.1.1. Structural Factors

Structural factors associated with IGD refer to relatively stable personal and/or biographical characteristics that shape vulnerability to problematic gaming.

(a) Demographic variables

Young adult males appear to demonstrate a greater propensity toward excessive video game use [25]. This pattern has been consistently observed across diverse populations of young adults in different academic settings across Europe, India, and the Arab region [26,27,28,29] and is often mediated by a predisposition toward dissociative experiences or driven by the gaming industry targeting male audiences [30].

Individuals who engage most extensively in gaming tend to be younger, less educated and underemployed. Among medical students a weekly gaming duration exceeding 20 h has emerged as a prominent predictor of PGB, with gender and psychopathology acting as moderating variables [31]. Furthermore, studies have identified a declining prevalence gradient across academic years in both general student populations [32] and medical student cohorts [23], with first-year students exhibiting the highest prevalence rates. A possible explanation, as suggested by Chiang et al.’s meta-analysis [23] is that the transition to university is highly stressful for first-year medical students due to increased academic and independent demands. Because stress is a known risk factor for Internet Gaming Disorder (IGD), this challenging transition period may explain the higher rates of IGD observed among younger medical students.

(b) Personality characteristics

They have been consistently associated with an increased risk of problematic gaming. These include introversion, hostility, impulsivity, higher neuroticism and low conscientiousness, whereas traits such as extraversion, openness, agreeableness, conscientiousness, self-control, self-esteem, life satisfaction, and higher academic achievement appear to exert protective effects [33,34,35,36]. Distinct gamer profiles, ranging from socially oriented “seekers” to achievement-driven “achievers,” correlate with specific personality trait configurations [37,38]. Students playing 7 or more hours weekly scored lower in conscientiousness. Meanwhile, those playing a greater variety of games (e.g., role-playing games, puzzle games, strategy games, platformers, action-adventure games, and simulation games) scored higher in openness [39].

In medical students, higher levels of neuroticism positively correlate with video game addiction, whereas agreeableness, extraversion, and conscientiousness demonstrate a negative correlation [33,40]. Notably, conscientiousness is the sole personality trait that exerts a statistically significant protective function against gaming pathology [33,41].

#### 1.1.2. Contextual Factors Associated with Videogaming

Contextual risk factors associated with problematic gaming among university students and young adults have been extensively documented across diverse cultural settings. Longitudinal findings identified both risk and protective factors, thereby supporting the conceptualization of IGD as a dynamic and potentially modifiable condition across the lifespan.

(a) Psychiatric comorbidity has important connections to gaming behaviour, with evidence suggesting complex and frequently bidirectional relationships [42]. Early investigations of internet addiction among college students identified depression, attention-deficit/hyperactivity disorder (ADHD), social phobia, and hostility as significant correlates [23,43].

Although problematic gaming has been associated with anxiety symptoms [44], evidence of anxiety as a direct etiological factor in gaming addiction remains limited. In fact, moderate gaming has been shown to alleviate anxiety, facilitate social connectedness, and improve mood [6,44]. Similarly, depressive symptomatology has been associated with both increased gaming engagement and adverse psychosocial outcomes [35,45]. Nevertheless, certain forms of gaming, particularly physically interactive “exergames,” can successfully reduce depressive symptoms [6,7,46].

These relationships appear especially interesting in the context of medical education. Compared with students enrolled in other academic programs, medical students experience longer periods of study and substantially greater academic and emotional demands which often may precipitate or exacerbate depression, anxiety, and burnout, potentially contributing to the elevated prevalence of addictive behaviours. A previous meta-analysis reported a pooled prevalence of internet addiction among medical students of approximately 30.1% (five times the general population), while the pooled prevalence of IGD specifically was estimated at 6.2% (twice the general population) [23]. This latter finding is particularly noteworthy, given that IGD diagnosis requires the endorsement of at least five DSM-5 criteria, suggesting that subclinical gaming-related difficulties may be considerably more prevalent. Supporting this interpretation, a recent cross-sectional study employing the IGDS9-SF among Indian undergraduate medical students found that IGD was significantly associated with insomnia, poor academic performance, and aggressive behaviour, thereby underscoring the clinical importance of screening for PGB [47]. Furthermore, a large-scale Indonesian study identified weekly gaming duration, male gender, and membership in gaming communities as primary contextual predictors of IGD among medical students, with psychopathological variables functioning as moderators [31].

(b) Social and interpersonal factors

The association between social and interpersonal factors and video gaming is multifaceted, given that video gaming may contribute to both beneficial outcomes [48] and adverse consequences for social relationships [49,50]. The global proliferation of video gaming and excessive gaming behaviour may precipitate a decline in interpersonal connectivity, manifest as diminished participation in familial, peer-based, and community activities [50].

Much less studied is how social variables (family structure and group dynamics, including social support and prosocial behaviour within them) can influence video gaming behaviour.

(b1) Family structure

Family represents a fundamental contextual determinant of gaming behaviour and psychological well-being, exerting a substantial influence on both vulnerability to and protection from problematic gaming. Inconsistent parenting practices, limited supervision, low emotional responsiveness, rigid and dysfunctional family structures have been linked to a range of addictive behaviours [51,52]. Major disruptions, including parental divorce, socioeconomic disadvantage, and parental mental health difficulties, may further impair developmental adjustment and increase reliance on gaming as a maladaptive coping strategy, while excessive gaming can in turn intensify familial strain and reinforce a cycle of relational disengagement. Consistent with this, long-term family conflict and low socioeconomic status have been associated with greater self-isolation and gaming involvement [53,54].

Oppositely, well-functioning family structures provide an important protective buffer against problematic gaming. High-quality parent–young adult relationships, characterized by emotional support, effective communication, and appropriate monitoring are particularly protective during transitions to university, when parental oversight typically decreases [35]. Population-based findings similarly indicate that perceived social support, and family connectedness are inversely associated with IGD severity [55]. While parental monitoring has been identified as a key protective factor across genders [56], overly restrictive or punitive parenting strategies—such as rigid rule enforcement and strict limitation of access—do not appear effective in reducing problematic gaming and may, in some cases, exacerbate symptoms, particularly among male students [57].

(b2) Peer-group dynamics have also been shown to influence PGB. Membership in peer groups characterized by maladaptive social influences has been associated with increased gaming time, loneliness, social media addiction, aggressive behaviour, anxiety, ADHD symptoms, depression, sensation seeking, social vulnerability, and impulsivity [35]. Although in-game communication may foster meaningful social connections through self-disclosure and reciprocity [58], these online relationships often lack the emotional depth and stability associated with offline interpersonal relationships and may displace real-world social interaction [59,60]. Moreover, lower perceived social support has consistently been identified as a predictor of IGD severity [42,59].
(b3) Finally, social support may serve as a mediator that mitigates the impact of stress and anxiety on IGD [61]. An important aspect revealed by previous research is that offline social support, including interactions with family and peers, has a stronger protective effect than online social interactions, and has the effect of reducing anxiety, stress, and depressive symptoms in students with IGD [62].

In academic populations, contextual factors (satisfaction with relationships with family and colleagues, involvement in social activities) are variables that affect mental health [63,64] and may increase vulnerability to IGD [65,66].

Taken together, the aforementioned findings delineate a consistent profile of risk factors associated with PGB; however, while these patterns provide a useful framework for understanding the phenomenon, evidence from Eastern European populations—and particularly Romania—remains limited, underscoring the need for further investigation. Recent Romanian data indicate substantial engagement in video gaming, with approximately 7.9 million individuals aged 15–64 participating and an estimated 11.4% prevalence of video game addiction among adolescents, particularly in specific educational subgroups [67,68]. However, existing evidence remains largely confined to prevalence estimates, with limited insight into the psychological determinants, risk mechanisms, and broader contextual correlates of IGD. In academic contexts, despite the growing scholarly interest in preserving students’ health and well-being, empirical research specifically examining PGB remains scarce. This gap in the literature constitutes a primary rationale for the present study.

### 1.2. Objective and Hypotheses

The present study aimed to investigate the relationships between IGD, structural factors (demographic characteristics, personality traits) and contextual psychosocial factors (psychiatric comorbidity—depression and anxiety symptoms, social and interpersonal factors—family structure and social support, and prosocial behaviour) in students from a Romanian medical university. The study was conceptually guided by the IGD framework, with PGB as the outcome, because participants were assessed using a self-report screening instrument rather than a structured professional diagnostic interview.

The investigated hypotheses were:

**H1.**  *PGB is significantly predicted by a combination of demographic characteristics, gaming-related variables, personality traits, psychological distress, perceived social support, and prosocial behaviour: (H1.a.): Structural factors: Demographic characteristics are expected to show associations with PGB, although their independent contribution may be limited after accounting for personality and gaming-related variables. (H1.b.): Gaming-related contextual factors: Higher daily gaming time and preferred game type, particularly competitive gaming, are expected to be significant predictors of PGB. (H1.c.): Personality traits: The Big Five personality traits are expected to be associated with PGB. Based on previous evidence, lower conscientiousness is specifically hypothesized to be associated with higher PGB severity, whereas the associations of extraversion, agreeableness, negative emotionality, and openness to experience are examined exploratorily, because previous findings have been less consistent. (H1.d.): Perceived social support and prosocial behaviour: Higher perceived social support and higher prosocial behaviour are expected to be negatively associated with PGB.*

**H2.**  *Psychological distress and PGB: Depressive and anxiety symptoms are expected to be positively associated with PGB and to contribute to gaming-associated symptom severity.*

## 2. Materials and Methods

### 2.1. Study Design and Procedure

The design of the study was cross-sectional, with a single administration of a series of standardized psychometric instruments.

Data collection was carried out between December 2023 and December 2025 using a convenience sampling strategy. The study was advertised through online communication channels dedicated to undergraduate medical students at the “Carol Davila” University of Medicine and Pharmacy (CDUMP) in Bucharest, Romania—the largest and one of the most prestigious medical schools of Romania. The questionnaire was administered via Google Forms. Romanian students completed the Romanian-language versions of the questionnaires, whereas international students completed the corresponding English-language versions. The invitation included a brief description of the study objectives, target population, eligibility criteria, and the voluntary nature of participation. Eligible participants were current undergraduate medical students enrolled at the Faculty of Medicine at CDUMP. As part of the questionnaire, participants reported their year of study (1–6), providing an additional verification that respondents belonged to the target population. No financial, academic, or other incentives were offered for participation.

The study was run in accordance with the World Medical Association Declaration of Helsinki and was approved by the CDUMP Institutional Review Board (no. 20738/21 July 2023). Anonymity was guaranteed and no email addresses or other identifying data were collected. A researcher (M.I.D.) was available by email, in case there were questions or any type of disagreement regarding the process of filling in the questionnaires and for those participants intending to withdraw from the study. Final results were included in an IBM-SPSS 26.0^®^ (IBM Corp., Armonk, NY, USA) database.

### 2.2. Participants

Participants were undergraduate students, both Romanian and international, undergoing their studies and training at CDUMP. Inclusion criteria were: being at least 18 years old, having the status of current undergraduate students in the abovementioned institution, having agreed to the informed consent form, having completed all study instruments. Although various study programs take place within CDUMP, only students from Faculty of Medicine were included in this study. The exclusion criteria were incomplete or inappropriate answers to the questionnaires, being underage, not having given their consent, and participants who specified that they do not play video games.

### 2.3. Instruments

After providing informed consent, all participants completed a set of questions collecting demographic information, including age, year of study, gender, area of origin, family structure, marital status, engagement in professional activities in addition to studying, and, where applicable, the type of employment contract associated with their job. The next section referred to their gaming habits, specifically to the age of starting gaming activity, favourite devices (smartphone, personal computer, laptop, tablet, video game console), type of internet connection (Integrated Services Digital Network, satellite, Digital Subscriber Line, cable, dial-up, broadband, mobile, wireless, hotspot), daily average time spent in gaming (less than one hour, 2–3 h, 4–5 h, more than 6 h), preference regarding the time of week to play (during the weekend, during the week or the same), preferred type of game (competitive, cooperative, individual-casual), average amount of money (RON) spent monthly on games in the last year, perceived social support from family and friends (on a scale from 1 to 10), and a subjective evaluation about the availability of a person they can rely on, regardless of the moment or problem (expressed as a dichotomic answer yes/no).

Following the collection of demographic information, participants completed a battery of standardized psychological instruments selected on the basis of previous empirical evidence supporting their psychometric adequacy and relevance to the study objectives.

#### 2.3.1. Big Five Inventory–2 Extra-Short Form (BFI-2-XS)

The Big Five Inventory–2 Extra-Short Form (BFI-2-XS) [69] was used to assess the five major personality dimensions: extraversion, agreeableness, conscientiousness, negative emotionality, and openness to experience. The instrument consists of 15 items rated on a 5-point Likert scale ranging from 1 (“strongly disagree”) to 5 (“strongly agree”). Previous studies have reported satisfactory internal consistency, with Cronbach’s alpha coefficients ranging from 0.74 to 0.92 [70]. The Romanian adaptation of the BFI-2-XS was used in the present study [71].

#### 2.3.2. Patient Health Questionnaire-9 (PHQ-9)

Depressive symptoms were assessed using the Patient Health Questionnaire-9 (PHQ-9) [72], with a self-report measure corresponding to the nine diagnostic criteria for major depressive disorder outlined in the DSM-5 [10]. Participants rated the frequency of each symptom on a 4-point Likert scale ranging from 0 (“not at all”) to 3 (“almost every day”). For descriptive interpretation, scores of 0–4 indicate minimal depressive symptoms, 5–9 mild symptoms, 10–14 moderate symptoms, 15–19 moderately severe symptoms, and 20–27 severe depressive symptoms. These categories reflect symptom severity and should not be interpreted as establishing a clinical diagnosis of major depressive disorder. The PHQ-9 represents the self-administered version of the PRIME-MD diagnostic instrument for common mental disorders and has demonstrated excellent internal reliability, with a reported Cronbach’s alpha of 0.89 [73]. In this study we used a validated Romanian version [74].

#### 2.3.3. Generalized Anxiety Disorder-7 Scale (GAD-7)

Anxiety symptoms were measured using the Generalized Anxiety Disorder-7 Scale (GAD-7) [75], a self-report instrument designed to screen for probable cases of gener-alized anxiety disorder and evaluate symptom severity. The scale comprises seven items rated on a 4-point Likert scale ranging from 0 (“not at all”) to 3 (“almost every day”), yielding a total score ranging from 0 to 21. Scores of 0–4 indicate minimal anxiety symptoms, 5–9 mild symptoms, 10–14 moderate symptoms, and 15–21 severe anxiety symptoms. These categories were used descriptively and do not constitute a clinical diagnosis. The GAD-7 has shown good internal consistency, with Cronbach’s alpha coefficients exceeding 0.82 [76]. In this study we used a validated Romanian version [77].

#### 2.3.4. Multidimensional Scale of Perceived Social Support (MSPSS)

Perceived social support was assessed using the Multidimensional Scale of Perceived Social Support (MSPSS) [78]. The MSPSS consists of 12 items measuring perceived support from three sources: family, friends, and a significant other. Responses are provided on a 7-point Likert scale ranging from 1 (“strongly disagree”) to 7 (“strongly agree”). For descriptive interpretation, mean scores of 1.0–2.9 were classified as low perceived social support, scores of 3.0–5.0 as moderate perceived social support, and scores of 5.1–7.0 as high perceived social support [78]. These categories were used only for descriptive purposes, whereas continuous MSPSS scores were retained in the inferential analyses. The instrument has demonstrated strong psychometric properties, including good validity and internal consistency, with Cronbach’s alpha coefficients ranging from 0.88 to 0.97 [79,80]. The validated Romanian version of the MSPSS was administered [81].

#### 2.3.5. Prosocialness Scale for Adults

Prosocial behaviour was evaluated using the Prosocialness Scale for Adults [82]. This 16-item instrument assesses prosocial tendencies across domains such as helping, sharing, caring, and empathic responsiveness to others’ needs and requests. Items are rated on a 5-point Likert scale ranging from 1 (“never/almost never true”) to 5 (“almost always/always true”). Previous research has reported excellent internal consistency, with Cronbach’s alpha values ranging from 0.903 [83] to 0.932 [84]. The Romanian adaptation of the Prosocialness Scale for Adults, available through Research Central, was used in the present study [85].

#### 2.3.6. Internet Gaming Disorder Scale—Short Form (IGDS9-SF)

PGB was assessed using the Internet Gaming Disorder Scale—Short Form (IGDS9-SF) [86]. The scale is based on the nine diagnostic criteria for IGD included in the DSM-5 [10] and is designed to evaluate the severity of gaming-related symptoms and their associated negative consequences. The instrument comprises nine items rated on a 5-point Likert scale ranging from 1 (“never”) to 5 (“very often”), referring to gaming activities occurring over the preceding 12 months. The IGDS9-SF has demonstrated good reliability, with reported Cronbach’s alpha values of approximately 0.87 [87]. The Romanian version of the IGDS9-SF was administered [88].

Regarding this test, although higher IGDS9-SF cut-off scores have been proposed for the identification of probable clinical cases, lower thresholds are commonly employed in non-clinical and exploratory research to identify individuals exhibiting elevated levels of gaming-related symptoms or PGB. This approach is consistent with the broader literature, in which problematic gaming is frequently operationalized through questionnaire-based thresholds, although the specific cut-off values vary across instruments and populations [89,90,91].

In the present study, a cut-off score of 24 was adopted to classify participants as exhibiting PGB. This threshold was selected based on both conceptual and empirical considerations. Conceptually, a score of 24 reflects moderate-to-frequent endorsement of IGD criteria, without necessarily indicating the presence of severe or clinically significant impairment. Empirically, this score corresponded to values above the 90th percentile of the sample distribution, whereas a score of 18 represented the 75th percentile. The selected threshold ensured an adequate subgroup size for statistical analyses, while preserving sensitivity and statistical power. Consequently, findings derived from this classification should be interpreted as reflecting PGB or elevated IGD symptomatology rather than a formal clinical diagnosis of IGD.

### 2.4. Data Analysis

The analysis included firstly a descriptive level, with calculation of minimum, maximum, mean, standard error and standard deviation of numerical variables (e.g., age, age of starting gaming activity). Frequencies were also determined for the nominal variables (e.g., year of study, gender, country of origin). Because several continuous variables showed departures from normality, medians, interquartile ranges, skewness, and kurtosis were also calculated, and distributions were examined using histograms and normal probability plots. Subsequently, multiple variable association tests were carried out between demographic data, gaming habits and the psychometric instruments results, while also comparing Romanian and international students. Group comparisons were performed using Pearson’s chi-square tests, independent-samples *t*-tests, one-way ANOVA, and Spearman’s rank-order correlations, as appropriate. Spearman’s correlations were used to examine associations between IGDS9-SF scores and continuous psychological variables because of the observed departures from normality. Correlations were calculated using pairwise-valid observations. Categorical predictors were not included in the correlation matrix and were entered into the regression models using appropriate indicator coding.

A sensitivity power analysis was conducted using G*Power 3.1 for the final multiple regression model. With 243 participants, 18 predictors, α = 0.05, and power of 0.80, the minimum detectable effect size was f^2^ = 0.088, indicating adequate power to detect effects approaching medium magnitude.

Hierarchical multiple regression analyses were conducted to examine the contribution of structural factors (demographic variables and personality traits) and contextual factors (gaming-related behaviours) to psychological outcomes. In the main model variables were entered in blocks as follows: (1) demographic variables, (2) personality traits, (3) gaming-related variables, and (4) perceived social support. An additional regression model was run to assess contribution of depressive symptoms (PHQ-9), anxiety symptoms (GAD-7), perceived social support and prosocial behaviour to PGB. The primary hierarchical regression model included the MSPSS total score as an indicator of overall perceived social support. To examine whether specific sources of support showed differential associations with IGDS9-SF scores, we performed a sensitivity analysis where the total score was replaced with the Family, Friends, and Significant Other subscales. The total score and its constituent subscales were not entered simultaneously into the same model because of their conceptual and statistical overlap. Before hierarchical multiple regression, multicollinearity was assessed using tolerance and variance inflation factors (VIF). Regression assumptions were further evaluated by visual inspection of standardized residual histograms, normal P–P plots, and scatterplots of standardized residuals against standardized predicted values. Standardized residuals and Cook’s distance were also inspected to identify potentially influential observations. Standardized regression coefficients (β), R^2^, adjusted R^2^, ΔR^2^, and multicollinearity diagnostics (tolerance and VIF) were reported for the hierarchical regression models.

All data analysis was performed using IBM SPSS Statistics version 26.0 (IBM Corp., Armonk, NY, USA).

For all calculations, the threshold of statistical significance was *p* < 0.05.

## 3. Results

A total of 261 students consented to participate in the study. Of these, six participants were excluded because they were under the age of 18, and one participant was excluded due to missing age information. Additionally, 11 respondents were not included in the final sample, because they reported not engaging in video gaming. Consequently, the final analytical sample comprised 243 participants.

### 3.1. Participant Characteristics

The mean age of the participants was 20.53 years (SD = 2.23; range = 18–36). Participants reported initiating video gaming at a mean age of 10.61 years (SD = 3.40; range = 3–24), suggesting early exposure to gaming activities. Among the 204 participants who reported spending money on video games, the average monthly expenditure during the previous year was 65.52 RON (SD = 283.99; range = 0–3000). The substantial standard deviation indicates considerable variability in spending patterns across participants.

With regard to sociodemographic characteristics, the sample was predominantly female (58.0%), primarily composed of individuals residing in urban areas (86.1%), and largely consisted of Romanian students (72.0%), with international students accounting for 28.0% of the sample. The majority of participants were preclinical students (years I–II), representing 71.19% of the sample, whereas only 28.81% of those enrolled were in their clinical years (years III–VI). Preclinical students focus on classroom-based foundational sciences, while clinical students transition to hands-on patient care and practical training in hospital wards. Most participants reported having siblings (69.1%) and were not engaged in paid employment at the time of data collection (94.6%).

Regarding relationship status, the majority of participants identified as single (61.7%). Smaller proportions reported being in a non-cohabiting romantic relationship (27.2%), cohabiting with a partner (8.6%), or being married (2.5%).

The descriptive characteristics of demographic continuous variables are reported in Table 1.

### 3.2. Gaming Behaviour

Gaming behaviour was generally characterized by low-to-moderate daily engagement. Most participants reported gaming for less than 1 h/day (59.3%), followed by 2–3 h/day (34.2%). Only a minority reported gaming for 4–5 h/day (4.9%) or more than 6 h/day (1.6%). The majority of participants reported gaming during weekends (77.4%), whereas weekday-dominant gaming was rare (4.9%) (Table 2).

With regard to gaming preferences, individual/casual games (43.2%) and competitive games (41.6%) were most commonly reported, whereas cooperative games (15.2%) were least preferred. A descriptive gender pattern was also observed, with male participants showing a greater preference for competitive gaming, whereas female participants more frequently were engaged in individual/casual games.

In terms of devices, gaming was predominantly mobile-based. The smartphone was the most frequently used gaming device (72.4%), followed by the PC (52.7%), laptop (51.9%), console (42.0%), and tablet (35.0%). The most frequently reported connection type was wireless internet (77.4%), followed by mobile data (38.3%) and cable (37.9%), whereas other types of connection were rare. Connectivity relied mainly on wireless internet, reflecting high digital accessibility.

The mean IGDS9-SF score was 14.99 (SD = 6.03; range: 9–38). Using a cut-off score of 24 on the IGDS9-SF, 25 participants (10.28%) were classified as displaying PGB. The distribution of IGDS9-SF scores was positively skewed (skewness = 1.34; excess kurtosis = 1.62). The median score was 13, with an interquartile range of 10–18. The observed scores ranged from 9 to 38; a score of 9 represents the theoretical minimum of the instrument because each of its nine items is rated from 1 to 5.

### 3.3. Psychological and Social Variables

Participants reported relatively high perceived support from both family and friends. On a 1–10 rating scale, the mean perceived family support was 8.40, while the mean perceived support from friends was 8.02. In addition, 96.7% of participants reported that they had at least one person they could rely on regardless of the situation.

Based on the MSPSS interpretive criteria described above, most participants reported high levels of perceived social support. Specifically, 70.8% indicated high significant other support, 70.8% reported high family support, 62.6% reported high friend support, and 71.2% reported high overall support. Mean scores on the MSPSS dimensions were 5.63 (SD = 1.66) for significant other support, 5.59 (SD = 1.5) for family support, 5.35 (SD = 1.43) for friend support, and 5.52 (SD = 1.32) for total support.

Regarding personality traits, the highest average score was for openness to experience (M = 10.87, SD = 2.25), followed by conscientiousness (M = 10.18, SD = 2.07) and agreeableness (M = 10.10, SD = 2.14). In contrast, lower average values were found for emotional stability (M = 9.45, SD = 2.60) and extraversion (M = 9.27, SD = 2.44).

Regarding depressive symptoms, the mean PHQ-9 score was 9.13 (SD = 5.70). Although 24.3% of participants reported no depressive symptoms and 33.3% were in the subthreshold range, 25.5% presented moderate symptoms and 16.9% had moderately severe or severe depressive symptoms. With respect to functional impairment associated with depressive symptoms, 56.0% reported that these symptoms made work, household tasks, or social interactions “somewhat difficult”, while 15.7% reported severe or extreme impairment.

The mean GAD-7 score was 8.19 (SD = 5.56). Minimal anxiety was the most frequent category (30.9%), followed by mild (29.6%), moderate (24.7%), and severe anxiety (14.8%).

The mean score for prosocial behaviour was 60.79 (SD = 13.64), suggesting an overall moderate-to-high level of self-reported prosocial tendencies in the sample.

Distributional characteristics and internal consistency estimated for the principal psychometric variables are further presented in Table 3. MSPSS scores showed negative skewness, reflecting the concentration of responses at relatively high levels of perceived support. PHQ-9, GAD-7, and most personality scores showed smaller variances from symmetry.

### 3.4. Factors Associated with PGB

Chi-square analyses were performed to examine associations between PGB and selected categorical variables. Significant associations were identified for family structure (*χ*^2^ = 4.647, *p* = 0.031), daily gaming time (*χ*^2^ = 15.283, *p* = 0.020), and preferred game type (*χ*^2^ = 10.897, *p* = 0.040). Participants with siblings, those reporting longer daily gaming time, and those preferring competitive games showed higher frequencies of problematic gaming (Table 4).

A significant association was also observed for student origin (*χ*^2^ = 7.976, *p* = 0.005). Specifically, international students exhibited a greater prevalence of problematic gaming compared to their Romanian counterparts. No statistically significant relationships were identified concerning year of study, gender, area of residence, marital status, or preferred gaming time. Perceived availability of support in difficult situations was not significantly associated with PGB (Fisher’s exact *p* = 0.586; Cramer’s V = 0.013. The complete exploratory results for all categorical predictors are presented in Supplementary Table S1.

#### Bivariate Correlations

Spearman’s rank-order correlations between the principal continuous variables are presented in Table 5. Higher IGDS9-SF scores were associated with greater monthly gaming expenditure (*ρ* = 0.240, *p* = 0.001), higher depressive symptoms (*ρ* = 0.255, *p* < 0.001), and higher anxiety symptoms (*ρ* = 0.247, *p* < 0.001). Higher problematic gaming scores were also associated with lower agreeableness (*ρ* = −0.166, *p* = 0.010), lower conscientiousness (*ρ* = −0.250, *p* < 0.001), lower perceived social support (*ρ* = −0.179, *p* = 0.005), and lower prosocial behaviour (*ρ* = −0.214, *p* = 0.001). No statistically significant correlations were observed between IGDS9-SF scores and age, extraversion, negative emotionality, or open-mindedness.

### 3.5. Hierarchical Regression Analysis

#### 3.5.1. Structural and Contextual Factors Associated with PGB

A hierarchical multiple regression was conducted to examine the contribution of structural factors, gaming-related variables, and perceived social support to PGB, operationalized as IGDS9-SF total score. Demographic variables were entered in Block 1, personality traits in Block 2, gaming-related variables in Block 3, perceived social support (total score) in Block 4 and prosocial behaviour in Block 5. The country variable was excluded from the regression because, after listwise deletion, all participants retained in the complete-case sample were Romanian students (N = 152). The final model was statistically significant, explaining 28.9% of the variance in IGDS9-SF scores (R^2^ = 0.289, F(20,131) = 2.657, *p* < 0.001). Demographic variables entered in Block 1 did not significantly predict PGB, ΔR^2^ = 0.062, *p* = 0.227. The addition of personality traits in Block 2 resulted in a modest but non-significant increase in explained variance, ΔR^2^ = 0.054, *p* = 0.139. However, the inclusion of gaming-related variables in Block 3 significantly improved the model, ΔR^2^ = 0.155, *p* < 0.001, representing the largest incremental contribution to IGDS9-SF scores.

The addition of total perceived social support in Block 4 produced a small, marginal increase in explained variance, ΔR^2^ = 0.017, *p* = 0.078, but did not reach conventional statistical significance. Finally, the addition of prosocial behaviour in Block 5 did not improve the model, ΔR^2^ < 0.001, *p* = 0.792.

In the final model, higher daily gaming time remained a significant positive predictor of PGB, *β* = 0.292, *p* = 0.001. Preferred game type was also a significant predictor, *β* = −0.232, *p* = 0.006. Given the coding of this variable, this result indicates that lower scores on the game-type variable, corresponding to competitive gaming, were associated with higher IGDS9-SF scores. Conscientiousness was a significant negative predictor, *β* = −0.201, *p* = 0.017, suggesting that lower levels of self-regulation, organization, and persistence were associated with higher PGB. Total perceived social support showed a negative but non-significant association with IGDS9-SF scores, *β* = −0.158, *p* = 0.142, and prosocial behaviour was not a significant predictor, *β* = −0.025, *p* = 0.792 (Table 6).

Multicollinearity diagnostics showed tolerance values ranging from 0.199 to 0.923 and VIF values ranging from 1.084 to 5.013. The highest VIF values were observed for age and year of study, reflecting their substantial overlap; the remaining predictors showed no evidence of problematic multicollinearity. Inspection of the regression diagnostic plots indicated some residual non-normality and non-constant variance. No observation had a Cook’s distance greater than 1, although several potentially influential observations were identified and the regression estimates were therefore interpreted cautiously.

The final hierarchical regression model explained 28.9% of the variance in IGDS9-SF scores, corresponding to a large overall effect (Cohen’s f^2^ = 0.406). Gaming-related variables made the largest unique contribution to the model (ΔR^2^ = 0.155; incremental Cohen’s f^2^ = 0.218), representing a medium effect, whereas the contributions of perceived social support (ΔR^2^ = 0.017; f^2^ = 0.024) and prosocial behaviour (ΔR^2^ < 0.001; f^2^ < 0.001) were small and negligible, respectively. Overall, problematic gaming behaviour was primarily associated with gaming-related behavioural characteristics, with a smaller contribution from personality traits and no independent contribution of perceived social support or prosocial behaviour.

An additional hierarchical regression model was conducted in which the total MSPSS score was replaced by the three MSPSS subscales (Supplementary Table S2). None of the three sources of perceived social support independently predicted IGDS9-SF scores: Significant Other (*β* = −0.087, *p* = 0.493), Family (*β* = −0.043, *p* = 0.657), and Friends (*β* = −0.067, *p* = 0.515). These findings indicate that the absence of an association between perceived social support and PGB was consistent regardless of whether social support was analysed as a total score or according to its individual sources.

#### 3.5.2. Additional Psychological–Social Model

Because depressive and anxiety symptoms were theoretically relevant but were not included in the main gaming-behaviour model, an additional sensitivity analysis was conducted to examine their contribution to IGDS9-SF scores alongside personality, social support, and prosocial behaviour. The results of the sensitivity hierarchical regression model are summarized in Supplementary Table S3.

In this regression model, IGDS9-SF total score remained the dependent variable. Demographic variables were entered in Block 1, Big Five personality traits in Block 2, depressive and anxiety symptoms in Block 3, the three perceived social support subscales (Significant Other, Family, and Friends) in Block 4, and prosocial behaviour in Block 5. The country variable was excluded from the analysis because it was constant in the analysed subsample (N = 165). Multicollinearity diagnostics indicated acceptable values for all predictors (tolerance = 0.215–0.897; VIF = 1.115–4.659), suggesting no problematic multicollinearity.

The final model was statistically significant, F(18,146) = 2.097, *p* = 0.009, explaining 20.5% of the variance in IGDS9-SF scores, although the adjusted R^2^ was modest, at 0.107. Demographic variables did not significantly contribute to the model, ΔR^2^ = 0.063, *p* = 0.165, and the addition of personality traits showed only a marginal contribution, ΔR^2^ = 0.060, *p* = 0.069. The inclusion of depressive and anxiety symptoms in Block 3 significantly improved the model, ΔR^2^ = 0.069, *p* = 0.002. However, the addition of the three perceived social support subscales (ΔR^2^ = 0.012, *p* = 0.545), and prosocial behaviour (ΔR^2^ = 0.001, *p* = 0.616), did not add significant incremental explanatory value. In the final model, Conscientiousness was the only significant independent predictor, *β* = −0.207, *p* = 0.013, indicating that lower conscientiousness was associated with higher IGDS9-SF scores.

These findings support the relevance of psychological distress and conscientiousness in relation to problematic gaming symptom severity, while suggesting that perceived social support and prosocial behaviour did not independently explain additional variance after controlling for demographic, personality, and distress-related variables.

## 4. Discussion

The current study offers an in-depth investigation of video gaming activity and its psychological associations among native Romanian and international students. The findings partially supported the proposed hypotheses, suggesting a differentiated and multidimensional framework integrating behavioural, personality, and social factors.

Overall, gaming behaviour in this sample was generally characterized by low-to-moderate engagement, with most participants reporting less than one hour of daily gaming. Notwithstanding this comparatively restricted exposure at the group level, a clinically significant subset (10.28%) demonstrated PGB, reinforcing the idea that maladaptive gaming behaviours might arise even in groups with generally moderate usage.

These findings align closely with prior study conducted among Romanian student populations. Rusu et al. indicated that personality factors are variably linked to gaming motivations and preferences [92]. Individuals exhibiting lower levels of openness and conscientiousness were more inclined to engage in gaming as a means of alleviating boredom, while those with strong extraversion indicated social motivations for gaming. Moreover, personality factors were associated with certain gaming preferences, with males favouring competitive genres and females opting for cognitively engaging or narrative-driven games.

The higher prevalence of PGB among male students is consistent with previous studies. This finding may reflect a combination of biological, psychological, and sociocultural factors, including gender differences in gaming preferences and stress-coping strategies. Male students may be more likely to use gaming as a means of stress relief or emotional regulation, whereas female students may be more likely to employ alternative coping mechanisms, such as seeking social support or engaging in other leisure activities. Additionally, the gender composition of medical schools should be considered when interpreting these findings, as it may influence the overall prevalence of PGB within the study population. Nevertheless, these explanations remain speculative, and further longitudinal research is needed to better understand the mechanisms underlying the observed gender differences.

The present study contributes to the existing body of research by demonstrating that, while personality characteristics are associated with gaming preferences and motivations, behavioural engagement, including time spent gaming and a preference for competitive games, is a more significant factor in the development of PGB. This suggests that personality may influence how individuals interact with games, whereas behavioural patterns determine the likelihood of maladaptive usage.

At the descriptive level, individuals indicated elevated levels of perceived social support, especially from family and close ties. Nonetheless, this did not completely protect against psychological distress, since a significant portion of subjects reported moderate to severe depression and anxiety symptoms. This indicates that simply having access of social support may be inadequate to mitigate emotional vulnerability. The correlation between gaming duration and problematic usage aligns with current literature, which recognizes excessive involvement as a key behavioural indicator of problematic gaming [87,93,94,95]. In the present study, even moderate increases in daily gaming time (e.g., 2–3 h) were associated with higher rates of problematic gaming, suggesting that risk may emerge at relatively low thresholds in vulnerable individuals.

The type of the game also played a significant effect. Individuals favouring competitive games had elevated levels of problematic gaming in contrast to those who preferred cooperative or relaxed games. This discovery aligns with prior studies emphasizing the reinforcing characteristics of competitive gaming settings, encompassing reward mechanisms, ranking dynamics, and social comparison processes [87,96].

Interestingly, family structure was associated with problematic gaming at the bivariate level, with higher rates observed among participants with siblings; however, this association did not remain significant in multivariate analyses. This finding may reflect shared gaming environments or peer-like social modelling within the family context [97], although current evidence on sibling influences in gaming behaviour remains limited and inconclusive.

At the inferential level, problematic gaming activity was correlated with multiple behavioural, structural, and psychosocial aspects. Bivariate analysis revealed correlations with gaming length, game type, family structure, financial investment, and psychiatric symptoms. Nevertheless, multivariate studies uncovered a more nuanced pattern, indicating that gaming-related characteristics served as the primary predictors, whilst personality traits assumed a secondary, still significant role.

Taken together, these findings support a three-layer model of gaming and psychological functioning.

First, problematic gaming appears to be primarily behaviour-driven, with engagement patterns, such as time spent gaming and preference for competitive games playing a central role. This is consistent with behavioural addiction models emphasizing reinforcement mechanisms, reward systems, and sustained engagement.

Second, personality factors appear to be more strongly associated with problematic gaming than prosocial functioning. Across the regression models, lower conscientiousness consistently emerged as an independent predictor of higher IGDS9-SF score. This data supports the idea that self-regulation, planning, persistence, and impulse control may be important protective variables in gaming-related outcomes. In the context of medical education, where students face high academic demands and increasing autonomy, lower conscientiousness may represent a vulnerability factor for difficulties in regulating leisure activities, including gaming. In the context of medical education, where students face high academic demands and increasing autonomy, lower conscientiousness may impair academic evolution (with poor studying, procrastination higher exam failure rates) and clinical performance (inability to manage in clinical settings), leading to unprofessional behaviours [98]. Also, lower conscientiousness may represent a vulnerability factor for difficulties in regulating leisure activities, with excessive gaming used as a coping strategy against academic stress.

Third, psychological distress was relevant in the supplementary psychological–social model. The inclusion of depressive and anxiety symptoms significantly improved the prediction of IGDS9-SF scores, suggesting that emotional distress is associated with problematic gaming symptom severity. However, in the final supplementary model, conscientiousness remained the only significant independent predictor. Therefore, this trend may reflect the significant overlap between depressive and anxious symptoms, as well as their mutual relationship with internal vulnerability. This shows that emotional discomfort may be part of a larger sensitivity profile linked with problematic gaming, rather than acting as a distinct predictor in this cohort. Given the cross-sectional methodology, these results should be viewed as association rather than directionality.

Fourth, in the context of problematic gaming, perceived social support appears to play a limited influence. Although lower levels of perceived support had small bivariate relationships with IGDS9-SF scores, social support did not provide substantial additive explanatory value in multivariate models. In the additional psychological–social model, the following addition of prosocial behaviour did not improve the model. Similarly, prosocial behaviour did not explain additional variance after demographic variables, personality traits, gaming-related characteristics, and perceived social support were controlled. Thus, the findings do not support the hypothesis that reduced prosocial functioning is a major independent predictor of problematic gaming in this cohort and is not consistent with literature which connects low social support to higher video gaming addiction risk [61,62,99]. However, there are studies that did not found significant relationship between the sub-dimensions of perception of social support and video gaming addiction [100] and a possible explanation of this discrepancy often stems from the support type: real-world support reduces the risk, whereas online or in-game support lacks the emotional depth to prevent problematic behaviour [60]. This supports a differentiated interpretation: problematic gaming appears to be mainly behaviour-driven and self-regulation-related, whereas prosocial behaviour appears to be more strongly embedded in personality and interpersonal support.

At the same time, the absence of an independent effect of perceived social support should not be interpreted as evidence that social factors are irrelevant. Rather, the findings may indicate that broad perceived social support is not sufficiently specific to capture the social mechanisms involved in problematic gaming. More specific variables, such as online versus offline support, gaming motivation, peer-group gaming norms, family monitoring, loneliness, academic stress, and the social function of gaming may better explain the relationship between social functioning and PGB.

These results have practical implications for medical students’ well-being. Screening for problematic gaming in medical students should not focus only on the number of hours spent gaming, but also on the type of gaming involvement, especially competitive gaming, and on students’ broader self-regulation capacities. PGB can be assessed using validated screening instruments, such as the IGDS9-SF, structured interviews exploring leisure activities and gaming habits, or screening questionnaires focusing on key behavioural indicators, including preoccupation, escapism, and functional impairment [101]. In addition, broader psychological assessment tools may help identify students experiencing psychological distress, maladaptive coping strategies, or difficulties with time management, all of which may be associated with problematic gaming behaviour. Further research is warranted to evaluate the utility of combining these assessment approaches for the early identification of at-risk students. Preventive interventions may benefit from addressing time management, coping strategies, sleep routines, academic stress, and awareness of highly reinforcing gaming environments. Interventions targeting problematic gaming should focus on behavioural regulation, including reducing excessive engagement and addressing reinforcement mechanisms associated with competitive gaming. In contrast, interventions aimed at reducing depression and anxiety should prioritize emotion regulation and vulnerability-related traits, particularly negative emotionality. Finally, interventions focused on strengthening supportive interpersonal environments may be more relevant for improving general psychosocial functioning than for directly reducing problematic gaming symptoms.

Future research should adopt longitudinal designs to clarify the temporal relationships between personality, gaming behaviour, and psychological outcomes. In addition, investigating potential mediating and moderating mechanisms would provide a more refined understanding of how these factors interact over time. Particular attention should be given to the qualitative aspects of social support, including its functionality and perceived adequacy, as these may be more relevant than its simple presence.

Overall, the present findings support a multidimensional and domain-specific perspective on gaming and mental health, emphasizing the need to move beyond one-dimensional models that uniformly link gaming behaviour to psychological outcomes.

## 5. Limitations

The study has a number of methodological limitations. The first limitation identified is that the sample used for the study was relatively homogeneous. The study sample was drawn from a single institution, with all participants enrolled in the same medical faculty. There was also a disproportion regarding the number of students in the preclinical years (I, II—173 students) and the clinical years (III, IV, V, VI—70 students). Moreover, participation in the study was determined by the voluntary decision of individuals through a self-selection process, generating a possible bias (the answers were provided by those with higher motivation or greater interest in the topic of the research) and a limitation of the generalizability of the results. Subsequently, the inclusion criteria eliminated the number of responses (some of the registered students were either minors or specified that they did not usually play) and the final sample size was 243. The sample size was sufficient for the proposed multivariable analyses; however, it cannot be regarded as representative for all Romanian medical students. Thus, the findings have limitations in their generalisability to medical students nationally or internationally, particularly in the light of possible differences across universities, academic programs and cultural settings. Another limitation is represented by the measurement of time spent in video gaming through self-reporting by the student and the lack of objective assessment of it. Estimation errors can also be found within other variables regarding gaming habits, such as the age at which the game was started or the average monthly amount spent for this purpose. Another possible source of error is represented by the psychological variables assessed using standardized question tests. Despite guaranteed anonymity, the use of self-report scales could lead to social desirability bias. This might be particular in medical students, which may alter their responses to align with perceived professional ideals and perfectionism, Thus, the prevalence of the studied phenomena may be underestimated. Students may have underreported symptoms of anxiety and depression and minimized the facts related to their gaming habits, and overreported prosocial behaviours and perceived social support. Furthermore, anxiety or depression reported by students can be influenced by various external social or academic factors, such as personal life, financial situation or stress associated with the exam session. Therefore, interpretation should be made with caution, as there are possible associations between these variables themselves and gaming behaviour.

While the IGDS9-SF scores were obtained from a self-report screening questionnaire and no professional diagnostic interview was conducted, the results should be viewed as reflecting the severity of problematic gaming symptoms rather than a formal diagnosis of Internet Gaming Disorder.

As expected in a non-clinical group in which most participants report relatively modest levels of problematic gaming, the IGDS9-SF total score was positively skewed. Regression diagnostics also pointed to some residual non-normality and heteroscedasticity as well as some potentially influential observations. Multiple linear regression is generally fairly robust to moderate violations of normality, especially in samples of this size, but these violations could have affected the precision of standard errors and significance tests. Thus, regression results should be regarded with caution, especially with reference to less frequent participant or gaming categories.

Several BFI-2-XS domain scores showed low internal consistency, particularly conscientiousness and agreeableness. Although very short scales may produce attenuated Cronbach’s alpha coefficients, the personality-related findings should therefore be interpreted cautiously.

Last but not least, the cross-sectional design does not allow judgements on temporal sequence and causality. The observed connections may be bidirectional, for example, psychological distress may contribute to problematic gaming, but problematic gaming may also exacerbate depressed or anxious symptoms. Future multicentre studies with broader and more representative sampling procedures and longitudinal or prospective designs are needed to confirm the findings and to further clarify them.

## 6. Conclusions

The current study found that PGB among medical students is mostly linked to gaming involvement and personality-related self-regulation. Higher daily gaming time, preferred game type, and poorer conscientiousness were the strongest predictors of IGDS9-SF results. These findings are especially important for medical students, who confront intense academic pressure, ongoing cognitive demands, and increased autonomy. In this setting, problematic gaming may indicate challenges with self-management and coping, which could have an impact on sleep, study routines, academic functioning, and psychological well-being.

Psychological distress increased IGDS9-SF scores at the block level, but depression and anxiety did not remain independent predictors in the final model. Perceived social support and prosocial behaviour had no significant explanatory value, implying that problematic gaming in this population was not largely caused by poor social or prosocial functioning. The data should be taken as indicating PGB or elevated IGDS9-SF symptom severity, rather than clinically diagnosed Internet Gaming Disorder. Future longitudinal studies should examine factors such as gaming motivation, sleep, academic stress, burnout, and coping strategies to further elucidate the complex relationships between gaming behaviour, psychological well-being, and adaptation among medical students.

## Figures and Tables

**Table 1:** Descriptive Characteristics of the Study Sample: Continuous Variables.

Variable		N	Min.	Max.	Mean	SD
Age		243	18	36	20.53	2.23
Age at gaming onset		243	3	24	10.61	3.40
Self-report of gaming expenditure	Yes (RON/month)	204	5	3000	65.52	283.99
	No	39	N/A	N/A	N/A	N/A

Note: N/A = not applicable.

**Table 2:** Descriptive Characteristics of the Study Sample: Categorical Variables and Gaming-Related Behaviours.

Variable	Category	*n* (%)
Gender	Male	102 (42.0)
	Female	141 (58.0)
Area of residence	Urban	209 (86.1)
	Rural	34 (13.9)
Student enrollment type	Romanian	175 (72.0)
	International	68 (28.0)
Year of study	Preclinical	173 (71.19)
	Clinical	70 (28.81)
Structure of the origin family	Single child	75 (30.9)
	With siblings	168 (69.1)
Relationship status	Single	150 (61.7)
	In a relationship, not cohabiting	66 (27.2)
	In a relationship, cohabiting	21 (8.6)
	Married	6 (2.5)
Employment status	Not employed	230 (94.6)
	Part-time/full-time employed	13 (5.4)
Daily gaming time	<1 h/day	144 (59.3)
	2–3 h/day	83 (34.2)
	4–5 h/day	12 (4.9)
	>6 h/day	4 (1.6)
Preferred gaming period	Mostly on weekends	188 (77.4)
	Mostly on weekdays	12 (4.9)
	No preference	43 (17.7)
Preferred game type	Competitive	101 (41.6)
	Cooperative	37 (15.2)
	Individual/casual	105 (43.2)
Main gaming device	Smartphone	176 (72.4)
	PC	128 (52.7)
	Laptop	126 (51.9)
	Console	102 (42.0)
	Tablet	85 (35.0)
Main connection type	Wireless	188 (77.4)
	Mobile	93 (38.3)
	Cable	92 (37.9)
Problematic gaming	No	218 (89.7)
	Yes	25 (10.3)

**Table 3:** Distributional characteristics and internal consistency of the principal psychometric variables.

Variable	N	Possible Range	Observed Range	Mean	SD	Median	IQR	Skewness	Kurtosis	Cronbach’s α
IGDS9-SF total	243	9–45	9–38	14.99	6.03	13.00	10.00–18.00	1.337	1.623	0.869
PHQ-9 total	243	0–27	0–26	9.13	5.70	8.00	5.00–13.00	0.600	−0.131	0.830
GAD-7 total	243	0–21	0–21	8.19	5.56	7.00	3.50–12.00	0.430	−0.689	0.912
Extraversion	243	3–15	3–15	9.27	2.44	9.00	8.00–11.00	−0.114	−0.193	0.526
Agreeableness	243	3–15	5–15	10.10	2.14	10.00	8.00–12.00	−0.002	−0.521	0.389
Conscientiousness	243	3–15	5–15	10.18	2.07	10.00	9.00–12.00	−0.081	−0.449	0.179
Negative emotionality	243	3–15	3–15	9.45	2.60	10.00	8.00–11.00	−0.217	−0.658	0.580
Open-mindedness	243	3–15	5–15	10.87	2.25	11.00	9.00–12.00	−0.065	−0.574	0.495
MSPSS—Significant Other	243	1–7 ^‡^	1–7	5.63	1.66	6.25	5.00–7.00	−1.323	0.909	0.941
MSPSS—Family ^1^	243	1–7 ^‡^	1–7	5.59	1.50	6.00	4.75–6.75	−1.196	0.622	0.945
MSPSS—Friends ^1^	243	1–7 ^‡^	1–7	5.35	1.43	5.75	4.50–6.50	−0.992	0.648	0.966
MSPSS total ^1^	243	1–7 ^‡^	1–7	5.52	1.32	5.83	4.92–6.50	−1.288	1.408	0.947
Prosocialness Scale total	243	16–80	16–80	60.79	13.64	64.00	53.00–70.00	−0.891	0.538	0.946

Abbreviations: PHQ-9 (Patient Health Questionnaire-9); GAD-7 (Generalized Anxiety Disorder-7 Scale); IGDS9-SF (Internet Gaming Disorder Scale—Short Form); IQR = interquartile range. ^‡^ MSPSS subscale and total values are reported as mean item scores. ^1^ MSPSS subscale and total values are reported as mean item scores.

**Table 4:** Significant bivariate associations between categorical variables and problematic gaming behaviour (IGDS9-SF ≥ 24).

Variable	*χ* ^2^	df	*p*
Family structure	4.647	1	0.031
Daily gaming time	15.283	3	0.0020
Preferred game type	10.897	2	0.0040
Student origin	7.976	1	0.0050

**Table 5:** Spearman correlations between problematic gaming and the main continuous study variables.

Variable	1	2	3	4	5	6	7	8	9	10	11	12
1. IGDS9-SF score	-											
2. Age	−0.059	-										
3. Monthly gaming expenditure	0.240 ^‡^	0.026	-									
4. Extraversion	−0.077	−0.101	−0.012	-								
5. Agreeableness	−0.166 ^‡^	0.036	−0.205 ^‡^	0.108	-							
6. Conscientiousness	−0.250 ^‡^	0.015	−0.136	0.150 *	0.189 ^‡^	-						
7. Negative emotionality	0.124	0.149 *	−0.051	−0.273 ^‡^	−0.109	−0.292 ^‡^	-					
8. Open-mindedness	−0.056	0.100	−0.021	0.273 ^‡^	0.170 ^‡^	0.082	−0.021	-				
9. PHQ-9 score	0.255 ^‡^	−0.006	0.041	−0.194 ^‡^	−0.098	−0.242 ^‡^	0.549 ^‡^	0.018	-			
10. GAD-7 score	0.247 ^‡^	−0.049	0.001	−0.171 ^‡^	−0.104	−0.141 *	0.573 ^‡^	0.025	0.734 ^‡^	-		
11. MSPSS total score	−0.179 ^‡^	−0.042	−0.138 *	0.147 *	0.167 ^‡^	0.119	−0.140 *	0.120	−0.242 ^‡^	−0.155 *	-	
12. Prosocial behavior score	−0.214 ^‡^	−0.021	−0.080	0.135 *	0.348 ^‡^	0.103	−0.006	0.161 *	0.031	0.021	0.321 ^‡^	-

Note. Values are Spearman’s rank-order correlation coefficients, ρ. N = 243, except for correlations involving monthly gaming expenditure, for which N = 204 because of missing data. Pairwise deletion was used. MSPSS = Multidimensional Scale of Perceived Social Support; PHQ-9 = Patient Health Questionnaire-9; GAD-7 = Generalized Anxiety Disorder-7; IGDS9-SF = Internet Gaming Disorder Scale—Short Form. * *p* < 0.05. ^‡^ *p* < 0.01.

**Table 6:** Predictors of PGB.

Block	Predictor	*β*	*p*	Tolerance	VIF
1	Gender	0.053	0.542	0.712	1.404
2	Conscientiousness	−0.201	0.017	0.789	1.268
3	Daily gaming time	0.292	0.001	0.795	1.258
	Preferred game type	−0.232	0.006	0.799	1.252
4	Total Perceived Social Support Scale	−0.158	0.142	0.473	2.112
5	Prosocial behaviour	−0.025	0.792	0.594	1.684

Final model: R^2^ = 0.289; adjusted R^2^ = 0.180; F(20,131) = 2.657, *p* < 0.001.

## Data Availability

Data in anonymized form is available upon reasonable request from the study authors. The dataset is not publicly available because the informed consent provided to participants and the Ethics Committee-approved study protocol emphasised the confidentiality of individual-level research data and that the publication of study results will be limited to aggregated data.

## Supplementary Materials

The following supporting information can be downloaded at: https://www.mdpi.com/article/10.3390/healthcare14152348/s1, Supplementary Table S1. Bivariate Associations Between Demographic and Gaming-Related Categorical Variables and Problematic Gaming Behaviour; Supplementary Table S2. Sensitivity analysis using MSPSS subscales; Supplementary Table S3. Psychological and Social Predictors of PGB.

## Abbreviations

The following abbreviations are used in this manuscript:

PGB	Problematic Gaming Behaviour
IGD	Internet Gaming Disorder
PHQ-9	Patient Health Questionnaire—9 items
GAD-7	General Anxiety Disorder—7 items
BFI-2-XS	Big Five Inventory–2 Extra-Short Form
