# Supplementary material for: Psychological Correlates of Problematic Gaming Behaviour in Medical Students: A Cross-Sectional Analysis of Structural and Contextual Factors
Source: Healthcare (Basel). 2026 Aug 1;14(15):2348. doi: 10.3390/healthcare14152348 (PMC13465105; doi:10.3390/healthcare14152348)
Supplement: Supplementary file 1 [file healthcare-14-02348-s001.zip › Supplementary Table S1.pdf]

Supplementary Table S1. Bivariate Associations Between Demographic and Gaming-Related Categorical Variables and Problematic Gaming Behavior

Variable	Test used	Statistic	df	p	Cramer's V
Smartphone	Pearson χ^2	0.003	1	0.960	0.003
PC		2.625	1	0.105	0.104
Laptop		0.688	1	0.407	0.053
Tablet		0.109	1	0.742	0.021
Gaming console		1.150	1	0.284	0.069
ISDN	Fisher exact	—	—	0.279	0.085
Satellite		—	—	0.274	0.066
DSL		—	—	0.354	0.063
Cable	Pearson χ^2	1.152	1	0.283	0.069
Dial-up	Fisher exact	—	—	1.000	0.038
Mobile	Pearson χ^2	0.035	1	0.851	0.012
Wireless		7.265	1	0.007	0.173
Hotspot		1.154	1	0.283	0.069

Note. Pearson's χ^2 test was used unless expected cell frequencies were <5, in which case Fisher's exact test (two-sided) was applied.
