# Supplementary material for: Psychological Correlates of Problematic Gaming Behaviour in Medical Students: A Cross-Sectional Analysis of Structural and Contextual Factors
Source: Healthcare (Basel). 2026 Aug 1;14(15):2348. doi: 10.3390/healthcare14152348 (PMC13465105; doi:10.3390/healthcare14152348)
Supplement: Supplementary file 1 [file healthcare-14-02348-s001.zip › Supplementary Table S2.pdf]

Supplementary Table S2. Sensitivity Analysis Using MSPSS Subscales

MSPSS subscale	β	p	Tolerance	VIF
Friends	−0.067	0.515	0.521	1.921
Family	−0.043	0.657	0.579	1.727
Significant Other	−0.087	0.493	0.343	2.919

Block 4 MSPSS subscales: $\Delta R^2 = .017$, $p = 0.374$.

Final Model: $R^2 = .289$; adjusted $R^2 = .168$; $F(22,129) = 2.381$, $p = 0.001$.
