# Supplementary material for: Psychological Correlates of Problematic Gaming Behaviour in Medical Students: A Cross-Sectional Analysis of Structural and Contextual Factors
Source: Healthcare (Basel). 2026 Aug 1;14(15):2348. doi: 10.3390/healthcare14152348 (PMC13465105; doi:10.3390/healthcare14152348)
Supplement: Supplementary file 1 [file healthcare-14-02348-s001.zip › Supplementary Table S3.pdf]

Supplementary Table S3. Psychological and Social Predictors of PGB

Block	Predictor	B	SE	β	p	Tolerance	VIF
1	Age	−0.477	0.502	−0.151	0.344	0.215	4.659
	Year of study	0.264	0.652	0.063	0.686	0.227	4.407
	Gender	−1.425	0.962	−0.125	0.141	0.765	1.308
	Area of residence	−0.762	1.306	−0.048	0.560	0.822	1.217
	Family structure	0.447	0.884	0.039	0.614	0.897	1.115
	Marital status	1.013	0.686	0.133	0.142	0.671	1.491
	Employment status	−2.266	2.562	−0.070	0.378	0.872	1.147
2	Extraversion	0.022	0.191	0.010	0.909	0.708	1.412
	Agreeableness	0.182	0.240	0.065	0.449	0.746	1.340
	Conscientiousness	−0.533	0.211	−0.207	0.013	0.811	1.233
	Negative emotionality	−0.333	0.218	−0.154	0.129	0.532	1.881
	Open-mindedness	0.179	0.201	0.071	0.376	0.847	1.181
3	GAD-7 score	0.175	0.122	0.177	0.153	0.356	2.806
3	PHQ-9 score	0.167	0.124	0.169	0.180	0.347	2.883
4	MSPSS—Significant Other	−0.340	0.416	−0.096	0.416	0.396	2.523
	MSPSS—Family	0.129	0.394	0.031	0.745	0.587	1.704
	MSPSS—Friends	−0.226	0.407	−0.054	0.579	0.582	1.718
5	Prosocial behavior	−0.021	0.041	−0.047	0.616	0.631	1.585

Model Summary by Block

Block	Predictors entered	R^2	ΔR^2	p for ΔR^2
1	Demographic variables	0.063	0.063	0.165
2	Big Five personality traits	0.124	0.060	0.069
3	PHQ-9 and GAD-7	0.192	0.069	0.002
4	Perceived social support (Significant Other, Family, and Friends subscales)	0.204	0.012	0.545
5	Prosocial behavior	0.205	0.001	0.616

Note. Dependent variable: IGDS9-SF total score. Values in the coefficient section are from the final model. N = 165. The country variable was excluded because it was constant in the complete-case subsample. The final model was statistically significant, $R^2 = 0.205$, adjusted $R^2 = 0.107$, $F(18,146) = 2.097$, $p = 0.009$. Tolerance values ranged from 0.215 to 0.897, and VIF values ranged from 1.115 to 4.659. B = unstandardized regression coefficient; SE = standard error; β = standardized regression coefficient; MSPSS = Multidimensional Scale of Perceived Social Support; PHQ-9 = Patient Health Questionnaire-9; GAD-7 = Generalized Anxiety Disorder-7.
